# Tuning Peptide-Based Hydrogels: Co-Assembly with Composites Driving the Highway to Technological Applications

**DOI:** 10.3390/ijms24010186

**Published:** 2022-12-22

**Authors:** Valéria Gomes, Sérgio R. S. Veloso, Miguel A. Correa-Duarte, Paula M. T. Ferreira, Elisabete M. S. Castanheira

**Affiliations:** 1 Physics Centre of Minho and Porto Universities (CF-UM-UP), University of Minho, Campus de Gualtar, 4710-057 Braga, Portugal; 2 Centre of Chemistry (CQUM), University of Minho, Campus de Gualtar, 4710-057 Braga, Portugal; 3 LaPMET Associate Laboratory, University of Minho, Campus de Gualtar, 4710-057 Braga, Portugal; 4 CINBIO, Universidad de Vigo, 36310 Vigo, Spain

**Keywords:** magnetic nanoparticles, metal nanoparticles, hydrogels, peptides, graphene, clay nanoparticles, liposomes, peptide-based composite hydrogels

## Abstract

Self-assembled peptide-based gels provide several advantages for technological applications. Recently, the co-assembly of gelators has been a strategy to modulate and tune gel properties and even implement stimuli-responsiveness. However, it still comprises limitations regarding the required library of compounds and outcoming properties. Hence, efforts have been made to combine peptide-based gels and (in)organic composites (e.g., magnetic nanoparticles, metal nanoparticles, liposomes, graphene, silica, clay, titanium dioxide, cadmium sulfide) to endow stimuli-responsive materials and achieve suitable properties in several fields ranging from optoelectronics to biomedical. Herein, we discuss the recent developments with composite peptide-based gels including the fabrication, tunability of gels’ properties, and challenges on (bio)technological applications.

## 1. Introduction

**Figure 1:**
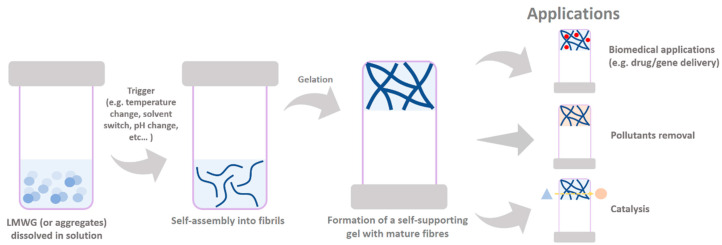
Cartoon schematic of LMWG self-assembly towards bulk gels and their wide-ranging applications. After a trigger, the LMWG molecules self-assemble into hierarchical structures (e.g., fibrils) that further progress towards entangled mature fibres with concomitant entrapment of the solvent to produce a gel. The gels can be employed in several applications, such as the delivery of drugs/genes entrapped in the gel matrix, the removal of pollutants through adsorption, or in catalysis if the gel fibres are endowed with catalytic units.

Hydrogels are highly useful soft materials in modern technological fields ranging from biomedicine to industry. There is a wide variety of gel types in nature, functionality, shape, and size, which mainly consist of elastic three-dimensional networks that retain a large amount of aqueous media [1,2]. This unique aspect provides a high physical similarity to tissues that drive the growing interest for its use in biological applications such as tissue engineering [3,4,5,6,7], wound healing [8,9], cell culture [10], biosensors [11], and therapeutics delivery [12,13,14]. Here, we focus on the use of peptide-based hydrogels, mainly the low-molecular-weight gelators (LMWG) that commonly display a size ranging from one to 20 amino acids [15]. These gelators self-assemble through a hierarchical assembly process into long anisotropic structures (e.g., nanofibers, nanotapes, nanotubes, or helical structures) that entangle or cross-link to form the gel matrix [16]. The assembly of LMWG is driven through the cooperative effect of specific intermolecular low energy interactions, such as hydrogen bonding, van der Waals, electrostatic, hydrophobic, and/or aromatic π-π interactions [17,18]. In general, the formation of gels is achieved through different triggers, such as pH, temperature, light, solvent-switch, ultrasound, enzymes, or addition of salt to a solution, or dispersion of the gelator molecules [19,20], which lead to a decrease in the molecule’s solubility and consequent aggregation (scheme included in Figure 1).

Besides this versatility of preparation, peptides are promising candidates for the development of soft materials owing to their biocompatibility, biodegradability, non-immunogenicity, similarity to the extracellular matrix, versatility, facility, stability, and robust structures that can be easily tailored and functionalized through amenable synthesis strategies, affording cost-effective gels with the required rheological properties [21,22,23,24,25,26,27]. In this sense, peptide-based gels are being progressively introduced in various technological fields, including optoelectronics, catalysis, and pollutant removal [28,29,30,31]. However, LMWG self-assembly commonly results in kinetically trapped states, as the self-assembly process is too fast for the structures to reach their targeted/global thermodynamic minimum [19]. A direct consequence is that different self-assembly pathways can have a profound impact on the final properties of the hydrogels [19,32]. For instance, some parameters that can have an influence are the temperature at which the gel is formed, the cooling rate in thermal annealing, the salt type and concentration, the final pH, or the solvent used. Although this may seem a hurdle to understand and rationalize the properties obtained from a large library of gelators, it provides a suitable means to achieve the desirable properties by tuning the self-assembly process of a smaller library. This large variety of possibilities on the fabrication and modulation of molecular gels based on peptides is well-discussed in various review articles that we suggest for the readers [20,33,34,35,36,37]. Recently, the tunability of gel properties took a step further by exploring the co-assembly of different gelators or non-gelators [38,39,40,41], which can not only widen the array of applications but also open the possibility of tailoring the material properties to specific application needs. The fabrication of multicomponent hydrogels includes the exploration of DNA-nucleobase-containing peptide derivatives [15,42], fabrication of materials with photopatterning capability [43], or even the control of properties through charge complementary gelators [44,45], which allow access to different gel states at a wide pH range. Adams’ group has been thoroughly advancing multicomponent gels and contributed reviews that we recommend to the readers interested in co-assembly of different LMWG [46,47,48], as we do not discuss these new materials.

Instead, we focus this review on the combination of peptide-based LMWG with non-gelling additives. In these systems, the composite can co-assemble with and/or adsorb to the hydrogel fibres or even remain in the aqueous phase and potentially induce a reinforcing or weakening effect on the gels’ mechanical properties (Table 1). The presence of composites can provide a powerful strategy to enhance the optoelectronic properties of LMWG-based gels, to enable unattainable functionalities, such as magnetic targeting and hyperthermia, photothermia, the compartmentalization of different drugs, or even the improvement of MRI contrast. Hence, we discuss the state of the art on the combination of peptide-based hydrogels with composites, their effect on the gels’ properties, and current challenges for future developments with these composite materials.

## 2. Liposome- and Niosome-Loaded Hydrogels for Drug Delivery

**Figure 2:**
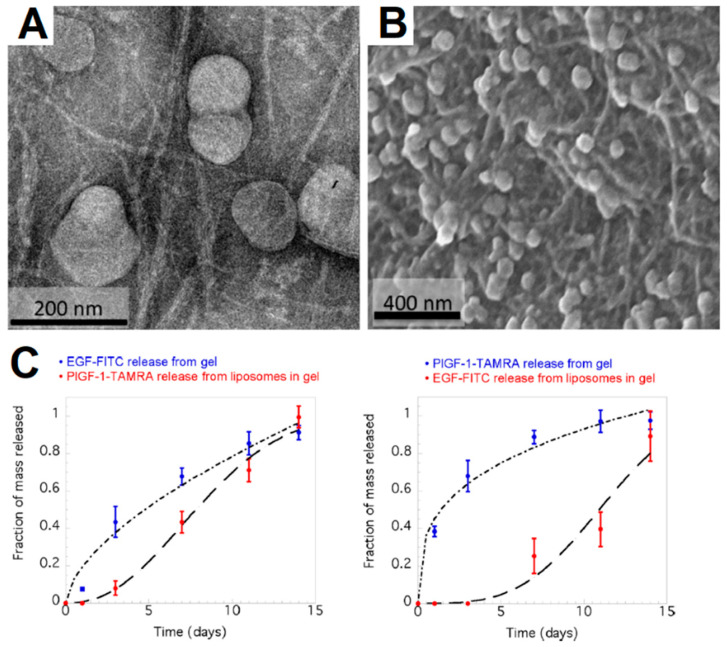
(**A**) Negatively stained transmission electron microscopy and (**B**) scanning electron microscopy images of peptide-based hydrogel containing liposomes. (**C**) Bimodal release profiles of two different growth factors (EGF-FITC and PlGF-1-TAMRA) either loaded in the hydrogel matrix (blue) or encapsulated in liposomes within the gel (red). Adapted from reference [61] with permission from American Chemical Society (ACS), 2022 (Notice to readers: further permissions should be directed to the ACS).

The combination of hydrogels and liposomes offers several advantages over the separate use of each system to the point of becoming a marriage of convenience, as titled in a review on liposome-encapsulated hydrogels by Grijalvo et al. [49]. The hydrogel matrix provides an efficient platform for the encapsulation and controlled release of therapeutic molecules, in which the passive drug release can be tailored through changes in the mesh size, composition, and crosslinking density of the network and interactions between network chains and the therapeutic molecules. However, like liposomes, a major aspect to consider in delivery applications is the initial burst release that can lead to drug accumulation and undesirable toxic effects [49,50]. The encapsulation of therapeutic drugs in liposomes and posterior incorporation in a hydrogel matrix can surpass this uncontrolled release through the combined transport resistance of the liposome membrane and hydrogel matrix [51,52,53,54], besides improving the liposome’s stability and loading of hydrophobic agents. The compartmentalization of therapeutic molecules further improves the spatial and temporal control over the system by enabling the on-demand release of compartmentalized (lethal) drugs, unstable molecules, or even the in situ synthesis of biologically active molecules from precursors when combined with a trigger [55]. However, there are few reports on the combination of peptide-based hydrogels with liposomes. In earlier works, liposomes loaded with calcium were explored as a means to induce the gelation of fibrinogen [56,57], or fibrin-derived gelators [58,59]. The strategies commonly employed liposome formulations that displayed a phase transition from the more solid phase (gel phase) to a more fluid phase (liquid-crystalline phase) at 37 °C [57], leading to the release of Ca^2+^ and consequent gelation, or the photo-responsive liposome formulations [56]. Recently, through the implementation of near-infrared responsive nanoparticles and thermosensitive liposomes in fibrin hydrogels, Martín-Saavedra et al. [60] obtained lipogels for on-demand release of doxorubicin. The authors demonstrated the DOX release dependence on the concentration of nanoparticles, the intensity of the electromagnetic energy and the irradiation time, and its bioactivity in cultures of epithelial carcinoma cells, whose properties were further improved by the use of copper sulfide nanoparticles and incorporation of cholesterol in the liposome formulations. However, these recent advances in hydrogels are still far from being achieved with the more complex molecular gels. In a work by Wickremasinghe et al. [61], hydrogels based on a multidomain peptide were loaded with liposomes in which a growth factor could be compartmentalized, and another growth factor would be encapsulated in the hydrogel matrix (Figure 2). The system provided a bimodal release profile that enabled the prolonged and sequential release of both loaded molecules. Later, the same strategy was employed by Majumder et al. [62] to sequentially deliver EGFR kinase inhibitor erlotinib (ERL) and doxorubicin (DOX) to synergistically kill glioblastoma, which is the most aggressive form of brain cancer.

Recently, our group demonstrated that both solid and aqueous magnetoliposomes could be incorporated into peptide LMWG-based hydrogels [63]. The interaction between the lipid-based system and hydrogel network was unaffected by the architecture, displaying a similar distribution of the hydrophobic drugs between the liposome membranes and hydrogel fibres. Regarding the design strategies of nanoarchitectures with peptide LMWG, Rosa et al. [64,65] demonstrated the importance of surfactants (including polyethylene glycol sorbitan monostearate and sorbitan monostearate) in the fabrication of nanogels based in dipeptides through different methodologies, which was also previously described by Gazit’s group in a top-down methodology [66]. Moreover, the authors demonstrated that the system could be used for the delivery of doxorubicin. Hereby, the advances taken in the combination of peptide-based hydrogels and liposomes demonstrate their promising use for biomedical applications.

Niosomes are vesicular nanocarriers comprising non-ionic surfactants, which have been increasingly employed as an alternative to liposomes for sustained and controlled drug delivery [67,68,69]. Like liposomes, the systems can be unilamellar, oligolamellar, or multilamellar, and the properties can be tuned by the addition of cholesterol and/or its derivatives and charged molecules. Moreover, niosomes are capable of encapsulating both hydrophilic and lipophilic molecules, in which the former are encapsulated in the inner aqueous core and/or adsorbed to the membrane, while the latter are localized in the lipophilic domain of the bilayer.

The niosomes also provide several advantages over their counterparts, mainly low cost of production, ease of formulation and scaling-up, and better physical and chemical stability achieved through the use of non-ionic surfactants compared to that of phospholipids commonly employed in liposomes [70]. Therefore, these systems have also been explored in combination with hydrogels to achieve improved controlled delivery of therapeutic drugs and avert undesired burst release effects [49]. However, the majority of the literature is dedicated to polymeric gels, being mostly devoid of supramolecular peptide-based gels. Recently, Grijalvo et al. [71] demonstrated the possibility of using a supramolecular hybrid hydrogel based on fluorenylmethyloxycarbonyl (Fmoc) *N*-protected phenylalanine and κ-carrageenan as a carrier for oligonucleotide-loaded nioplexes, which displayed better transfection efficiencies than unformulated hydrogels. Therefore, niosomes provide a suitable strategy for the development and optimization of hydrogels as tuneable delivery systems for drugs and nucleic acids.

## 3. Turning Hydrogels into Magnetic Materials

Magnetic hydrogels have been of great interest owing to the combination of both the hydrogel and magnetic nanoparticle advantages providing a suitable platform for a wide range of applications. For instance, the magnetic nanoparticles enable real-time remote control over various properties of the gels, from the micro- to the macroscopic scale, such as the shape, size, mechanical behaviour, and diffusion of encapsulated molecules [72,73]. Moreover, the nanoparticles allow the use of magnetic hyperthermia and targeting, which can synergistically enhance the control of the delivery of loaded molecules. The reader is referred to various reviews on magnetic gels that discuss the applications, as well as the progress of these materials to the more complex magnetic lipogels [55,72,74,75,76]. Regarding the use of peptide LMWG-based magnetic hydrogels, Table 2 summarizes some of the works discussed here.

In an early work by Yang et al. [77], the authors fabricated magnetic nanoparticles functionalized with DA-L-Phe-L-Phe-OH, which could co-assemble with the hydrogelator 2-Naph-L-Phe-L-Phe-OH in the fibre formation. The system displayed a fast magneto-mechanical response, which could not be achieved with nanoparticles, only functionalized with DA. Additionally, through the use of DA, Das et al. [78] reported the formation of magnetic hydrogels from the co-assembly of polydopamine spheres decorated with iron oxide nanoparticles with the peptide diphenylalanine, while the co-assembly of polydopamine particles with diphenylalanine resulted in tubular structures decorated with the magnetic nanoparticles. The authors believe that, in the former case, the interaction between magnetic nanoparticles and diphenylalanine occurs through the amine groups of this peptide, while XPS analysis revealed that, in the latter situation, the interaction between the polydopamine spheres and diphenylalanine occurs through the catechol groups on the tubular wall. This work demonstrates the wide variety of materials that can be obtained with small changes in composition and the complexity of the structures resulting from co-assembly. Another strategy was described by Contreras-Montoya [73] that included the use of PEG-coated nanoparticles covered with the hydrogelator. Through this particle design, anisotropic magnetic gels could be fabricated upon application of a DC magnetic field during gelation. Later, the same group applied a similar particle design strategy in a co-assembled hydrogel containing one peptide with RGD moiety to obtain stable gels that can be used as 3D scaffolds for cell growth [81]. The mechanical properties of the magnetogel provide cellular protection against shear forces, allowing it to be injected into tissues without causing relevant cell damage. Recently, Nowak et al. [80] simplified the co-assembly of nanoparticles with the gel fibres through the addition of aspartic acid to the C-terminal of the hydrogelator, which enabled the coordination of nanoparticles’ metal ions. In this way, the gel displayed an increased elasticity in the presence of the nanoparticles, besides providing a magneto-mechanical response at low nanoparticle concentration. Regarding the use in drug delivery, we reported in an earlier work that magnetic hydrogels could be used for the encapsulation of hydrophobic drugs, including curcumin and thienopyridine derivatives [84]. Moreover, the encapsulated drugs’ release could be enhanced through the use of photothermia using different nanoparticles architectures, core/shell manganese ferrite/gold nanoparticles, and gold-decorated manganese ferrite nanoparticles with ca. 55 nm and 45 nm sizes, respectively [82]. Recently, our group reported how different functionalization could impact the co-assembly and final properties of the gel [83]. Citrated-stabilized nanoparticles were observed to aggregate upon gelation, while lipid-coated nanoparticles tended to co-assemble along the hydrogel fibres. Nonetheless, both assemblies could induce the release of doxorubicin upon application of a low frequency alternating magnetic field. Although both assemblies presented low frequency magnetic field-induced doxorubicin release, this work shows that these composites can be deeply tuned and changed (e.g., in terms of architecture, mechanical properties, and drug encapsulation capacity) with simple modifications, owing to the great diversity of interactions existing on the molecular scale.

## 4. Composite-Loaded Gels for Tissue Engineering: Silica and Clay Nanoparticles

The inclusion of silica nanoparticles holds various advantages in biomedical applications. For instance, Becerra et al. [85] incorporated silica particles functionalized with chitosan in fibrin-based gels, which strongly improved the mechanical properties of the gel at a low composite concentration. This reinforcement effect of the mechanical properties was also explored by Cheng et al. [86] on silk fibroin (SF) hydrogels for bone tissue engineering. The presence of silica nanoparticles can provide different functions, such as: stimulate osteoblast mineralization and enhance bone mineral density; release bioactive silicon ions that are essential for bone metabolism; and partially mimic the distribution of mineral crystals in the extracellular matrix through the presence of partially aggregated nanoparticles in the hydrogel network. Through the combination of the described effects, the authors were able to improve the mechanical properties and osteogenic abilities of the gel, which provided a biocompatible microenvironment for cell adhesion, proliferation, and osteogenic differentiation. The reinforcement of hydrogels was also achieved by Cao et al. [87] through an amyloid-assisted biosilicification process that leads to the formation of silicified nanofibrils that can also be explored to fabricate stable aerogels. Besides the reinforcement capabilities, the combination of peptide-based hydrogels with silica particles has also been implemented in the development of Boolean logic gates (OR and AND), which respond to a specific assortment of proteases (e.g., chymotrypsin and trypsin) as reported by Ayyub et al. [88]. Here, peptide-based hydrogel-silica nanoparticle composites became stronger when subjected to the action of proteases. The key to this phenomenon was in the electrostatic forces (physical interactions) created between the terminal amine (generated after the catalytic cleavage of the peptide) and the surface of the negatively charged silica nanoparticles. Nonetheless, the possibilities with the combination of silica and peptides are diverse, be it the use of peptide-based materials as a template for the synthesis of silica nanostructures, as reviewed by Bellotto et al. [89], or in evaporation-induced self-assembly of nanoparticles [90]. Other promising nanostructures are the mesoporous silica nanoparticles, which were described by Baumann et al. [91], on the fabrication of injectable gel scaffold based on the self-assembling peptide RADA16-I, also loaded with MC3T3-E1 osteoblast precursor cells, for the development of a strategy for tissue engineering. Indeed, the multiple functionalities of mesoporous silica make it an asset in bone regeneration. However, the respective peptide composites lack controlled cargo release mechanisms (for tissue engineering) that match the dynamics of the tissues in concern.

**Figure 3:**
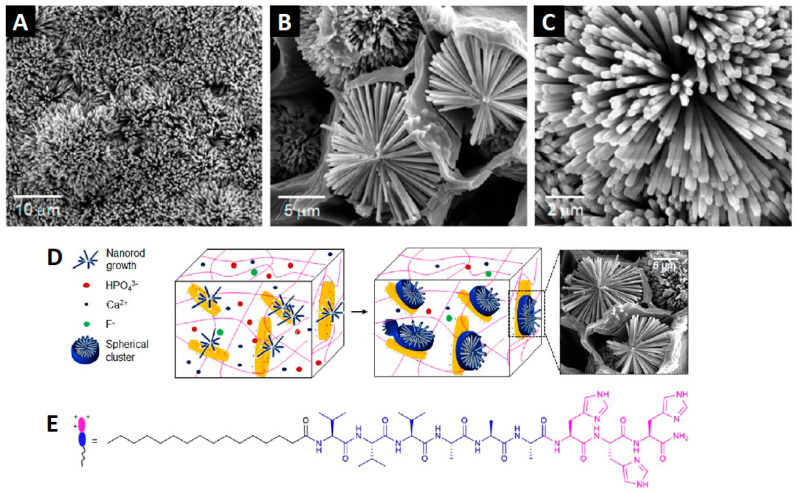
SEM images showing (**A**) dense crystal formation on the surface of a histidine peptide-based clay-hydrogel composite, which are organized into (**B**,**C**) spherical clusters of nanorods within the cavity of the hydrogels. (**D**) The hydroxyapatite crystals are formed upon the diffusion of the mineralizing ionic species into the histidine-based amphiphile peptide hydrogel loaded with Laponite^®^, which triggers the nucleation and hierarchical crystal growth of hydroxyapatite nanorods organized in spherical clusters. (**E**) Chemical structure of the peptide gelator. Adapted from reference [99].

In addition to the materials discussed so far, it is possible to include clay and layered silicates in the gels. These particles have long been studied and are easy to obtain. Interestingly, they develop plasticity when in wet environments and become hard upon drying [92,93]. Combining them with pristine hydrogels, producing composite gels, gives the latter benefits in terms of mechanical, thermal, optical, and physicochemical properties [94]. The most common clays employed in such composites are smectite clays, e.g., montmorillonite, saponite, and hectorite (e.g., Laponite^®^) [95,96,97,98]; however, the use of peptides as hydrogelators is still poorly explored, and the existing literature is quite recent. In 2021, for example, Okesola and co-workers [99] attempted the co-assembly of Laponite^®^ nanodiscs and histidine-based amphiphilic peptides, using lysine and glutamic acid analogues as controls. The structures resulting from electrostatic interactions between Laponite^®^ and the peptides allowed the hierarchical growth of hydroxyapatite into well-defined clusters (Figure 3). Furthermore, the histidine-based mineralized composites showed potential for tissue engineering, considering the enhancement of cell adhesion, proliferation, differentiation, and the neovascularization process in the chicken chorioallantoic membrane [99].

In another work, the electrostatic interactions between a positively charged L-arginine dendrimer and the negatively charged sodium polyacrylate-linked clay nanosheets produced a thixotropic hydrogel [100]. The mechanical properties and porosity of the supramolecular composite were found to be tunable according to the dendrimer content; antifouling capability was verified, making that material promising for medical devices, wound healing, and drug delivery applications [98]. Further work on these systems reports the use of peptides as co-factors for reinforcing and incorporating bioactive epitopes into the hydrogel network [101,102]. Such is the case of a hyaluronic acid tyramine-based hydrogel-clay composite that integrates the amphiphilic peptide C_16_H_31_CO-Val-Val-Val-Ala-Ala-Ala-Glu-His-Lys-Cu^2+^ and Laponite^®^ (used as a rheology modulator) [101]. The system promoted cell growth as well as osteoblastic differentiation and angiogenesis without exogenous growth factors [101]. Elsewhere, to assist the healing process of skin burns, Sorouri’s group incorporated carnosine (β-Ala-His-OH) and bentonite clays into a polymeric carbopol network [102]. The appropriate functional groups and the hydrophilic character of carnosine–bentonite combination endowed the gel with better viscoelastic properties compared to pure carbopol hydrogel—possibly as a consequence of the interactions between the imidazole and amine rings of carnosine with the carboxylic acid functions of carbopol. The authors demonstrated the effectiveness of the composite hydrogel in accelerating wound closure and re-epithelization, as well as in reducing inflammation relative to the pure carbopol hydrogel, making it interesting for topical applications [102].

## 5. Gold/Silver Nanoparticle-Loaded Gels for Biomedical and Environmental Applications

**Figure 4:**
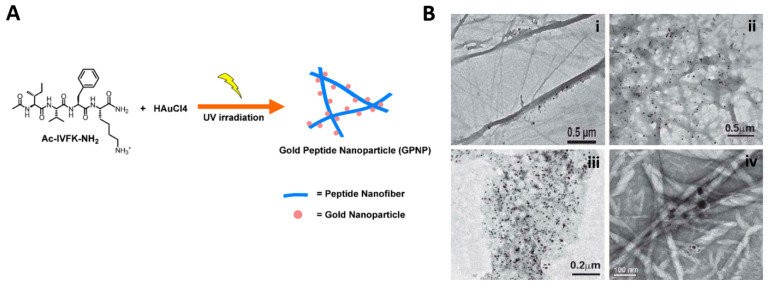
(**A**) Chemical structure of the tetramer peptide and fabrication of AuNPs composites with 254 nm light (adapted from reference [110]). (**B**) TEM images of AgNPs synthesized within self-assemblies of amphiphiles (**i**) ^+^(H_3_C)_3_-Trp-CONH-C_16_H_33_, (**ii**) ^+^(H_3_C)_3_-Tyr-CONH-C_16_H_33_, (**iii**) C_15_H_31_-CO-Val-Trp-COO^−^ and (**iv**) C_15_H_31_-CO-Ile-Trp-COO^-^. Adapted from reference [108], with permission from Royal Society of Chemistry, 2022.

Noble metal nanoparticles, such as those composed of gold (AuNPs) and silver (AgNPs), exhibit impressive physical–chemical properties and great resistance to harsh environments. They have been widely investigated for diagnostics, used as vehicles in drug delivery, and as thermal ablation enhancers in radiotherapy [103,104]. Combining them with hydrogels avoids their leaching, thanks to the immobilization of the nanoparticles in the matrix, which may enhance the functionalities of both materials and makes it possible to adjust several properties, such as the mechanical strength and bioactivity of the system [105]. Although the development of these composites is on the rise, the use of peptide building blocks is still largely unexplored [89,105,106]. To the best of our knowledge, most of these systems have peptides as gelators to modulate the synthesis of nanoparticles during or after hydrogel formation [89,105,107,108]. In the case of AuNPs-hydrogel composites, applications range from sensing to catalysis. The sophisticated work of Wang et al. [109] is an excellent example. They constructed a platform composed of a hydrogel based on the short peptide Fmoc-Phe-Glu-Lys-Phe-OH loaded with the antibiotic ciprofloxacin and AuNPs. The system, in addition to having shear-thinning behaviour and being biocompatible, demonstrated remarkable bacteriostatic activity—due to ciprofloxacin—and conductivity—resulting from AuNPs—which allowed it to be used as a catalytic dopamine sensor [109]. In a very recent work, Abbas and collaborators [110] developed a composite in which the Ac-Ile-Val-Phe-Lys-NH_2_ peptide forms fibres that serve as a template for the formation of gold-peptide nanoparticles by UV light action alone (Figure 4A). The authors explain the intervention of phenylalanine and lysine residues in the reduction of gold and highlight the fundamental role of lysine free amine in the nucleation and arrangement of nanoparticles by non-covalent interactions. The system showed very interesting catalytic activity in reducing small pollutants to less toxic compounds, which makes it promising for environmental technology applications [110]. It should also be noted that the conjugation of peptides with AuNPs is extremely advantageous, not only in the formulation of AuNPs-hydrogels, but also in addressing the major limitation of the latter, particularly in physiological environments, i.e., poor colloidal stability [111]. On the other hand, the work conducted so far on AgNPs-hydrogel composites focuses on the development of antibacterial materials [112,113,114,115,116] and the detection of analytes by surface-enhanced Raman scattering (SERS). Regarding this, in a novel work, AgNPs were synthesized in situ on different supramolecular fibre networks based on amphiphilic tryptophan and tyrosine peptides (Figure 4B) [108]. Interestingly, peptide gelators with different hydrophobicity resulted in different AgNPs’ synthesis times, leading, consequently, to a distinct level of antibacterial activity against Gram-positive and Gram-negative bacteria (Figure 4B) [108].

In another strategy, Almohammed et al. [117] used a minimalistic peptide gelator (Fmoc-Phe-Phe-OH) and combined it with AgNPs to serve as a low-cost 3D-printed SERS substrate. The system turned out to be capable of detecting molecules at picomolar concentrations, owing to the potentiation effect of the analytes’ Raman signal by the peptide fibres [117]. Interestingly, the authors suggest that the good performance of the sensor results both from the supramolecular interactions between the amine and/or carboxylic acid of the peptide hydrogelator and the nucleotide bases (improving the molecular association of the analyte) and from the possible pyroelectric properties of the former (making charge transfer easier when heated by the excitation laser). This work proves, once again, the possibility of manufacturing a standardized, aligned, low-cost, and highly sensible diagnostic platform through the development of this type of composite. Table 3 summarizes the advances made in these types of assemblies. Overall, we highlight the need for further studies regarding dispersion, uncontrolled release, reproducibility of the in situ synthesis, and long-term cytotoxicity (in the case of medical applications) of the peptide-embedded nanoparticles.

## 6. Carbonaceous Composites for Biomedical and Environmental Applications

**Figure 5:**
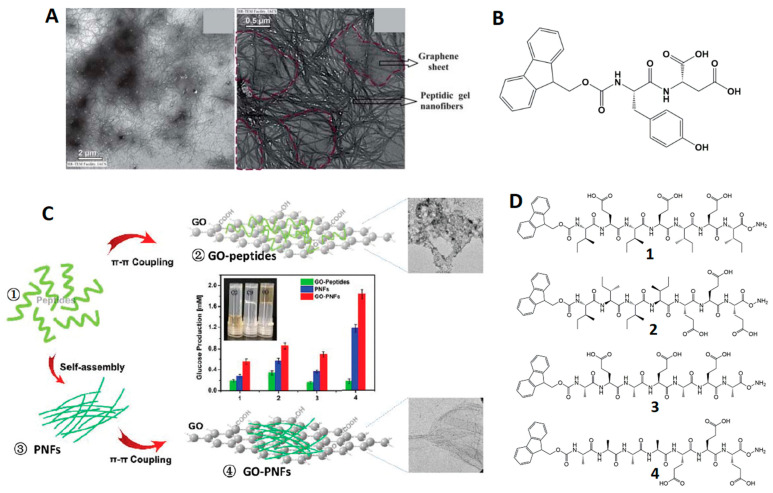
(**A**) TEM images of reduced graphene oxide-containing hydrogel obtained from (**B**) hydrogelator Fmoc-Tyr-Asp-OH. Adapted from reference [127] with permission from Royal Society of Chemistry, 2022. (**C**) Glucose production from the mixture of graphene oxide and peptides (GO-peptides), peptide hydrogels (PNFs) and graphene–peptide hydrogel composites (GO-PNFs); Adapted from reference [136] with permission from Royal Society of Chemistry, 2022. (**D**) Chemical structure of the peptide 1: Fmoc-Ile-Glu-Ile-Glu-Ile-Glu-Ile-CONH_2_, 2: Fmoc-Ile-Ile-Ile-Ile-Glu-Glu-Glu-CONH_2_, 3: Fmoc-Ala-Glu-Ala-Glu-Ala-Glu-Ala-CONH_2_, 4: Fmoc-Ala-Ala-Ala-Ala-Glu-Glu-Glu-CONH_2_.

Graphene, graphene oxide (GO), and its derivatives (carbon nanotubes, graphene quantum dots, etc.) have been intensively studied in the scope of electronics and biomedicine due to their electrical and thermal conductivities and transmittance [120]. Interest in these materials has emerged, in part, from the possibility of combining them with hydrogels forming advanced composites [121,122,123]. In addition to the many functional properties, graphene oxide, particularly, is highly advantageous in increasing the stability of supramolecular fibrillar networks—either in the dry or swollen state [120]—considering the assistance of the surface functional groups in phenomena such as self-assembly and gelation [121,124]. Although multiple applications are envisaged for these composites, some fundamental works are also reported, exploring the coexistence of materials that are so different [106] in porosity and mechanical behaviour [125,126,127]. For example, Adhikari et al. synthesized the hydrogelator Fmoc-Tyr-Asp-OH, which proved capable of incorporating GO sheets (Figure 5A,B) [127]. The π-stacking between the peptide fibres and carbon structures was used as a strategy for dispersing the latter (highly hydrophobic) in water, thus dispensing external stabilizing agents [127]. On the other hand, the applications are mainly focused on drug delivery, where graphene regulates drug release just by improving the rheological properties and controlling hydrogel erosion rate [128,129]. In an ambitious work, Schneible and co-workers developed a hydrogel based on the 20-residue peptide Max8 carrying the anticancer drug gemcitabine and doxorubicin-loaded GO nanoparticles [130]. The goal was to release that anticancer drug pair with defined kinetics and molar ratio in a synergistic and controlled way. It showed not only the increased synergy of the two drugs with respect to their free form in solution, but also with respect to their combination in microfluidic devices and engineered materials [130]. In the same context, Guilbaud-Chéreau et al. [131] incorporated GO and carbon nanotubes into gels based on Fmoc *N*-protected aromatic amino acids and used ascorbic acid as a model drug. Irradiating these systems with near-infrared (NIR) light promoted the diffusion of the drug into the medium because of the heat generated by the carbon materials, making these composites highly promising in the field of controlled drug and gene delivery [131]. By now, the reader must have noticed the great advantage of using low-molecular-weight peptides in the construction of the most diverse composites. The work of Guilbaud-Chéreau illustrates the freedom from covalent interactions—better for biomedical applications (due to injectability and responsiveness to biological stimuli), possibility of large-scale synthesis, and low cost. Regarding tissue engineering, the use of graphene–peptide hydrogel composites is still taking the first steps, with the regeneration of the nucleus pulposus being of note [132,133,134]. In a novel approach, the peptide Phe-Glu-Phe-Lys-Phe-Glu-Phe-Lys-OH was gelled at non-physiological pH (acidic and basic) in the presence of GO [132]. In addition to increasing the stiffness of the gels, the inclusion of graphene decreased the inflammatory response of nucleus pulposus cells, especially those encapsulated in the acidic composites (due to the greater interaction of GO with the fibres) [132]; this offers new perspectives in 3D cell culture. Nevertheless, the utility of peptide–graphene hydrogels extends from therapy to diagnostics and environmental catalysis [135,136]. Indeed, the production of environmentally friendly fuel from biomass requires the use of enzymes that are difficult to isolate [137]. To address this problem, He et al. [136] developed different platforms mimicking the enzyme β-glycosidase from glutamate-based peptide hydrogels loaded with GO nanosheets. Embedding graphene into the gel potentiated and accelerated the hydrolytic activity of the peptides (Figure 5 C,D) by accelerating proton transfer, which makes the designed systems efficient β-glycosidase mimetics under mild reaction conditions [136]. Table 4 summarizes the recent advances in peptide-based hydrogel–graphene composites.

## 7. Composites for Electronic, Catalytic, and Environmental Applications

Metal-oxide semiconductors have been increasingly employed for several environmental applications. Particularly, titanium dioxide nanoparticles, in the anatase form, have been of interest for photocatalytic degradation of organic pollutants, owing to its high catalytic activity and resistance to light corrosion, as well as an eco-friendly, scalable, and cost-effective synthesis [140,141,142].

Recently, Guidetti et al. [142] combined these nanoparticles with peptide-based hydrogels as a means to achieve efficient photoactive materials for water treatment. The authors reported a material with good transparency to solar light, in which the light penetrated and led to photodegradation of a small pollutant freely diffusing in the water channels of the material. The advantageous combination of TiO_2_ and peptide-based hydrogels was also demonstrated in the synergistic improvement of photoswitching properties (photocurrent gain) of the composite gel compared to the individual components, which is highly desired for the development of optoelectronic devices with high photocurrent conversion efficiency [141].

Besides the blending of hydrogel’s matrix with particles, the peptide’s fibres can also be used as templates for the nanofabrication of novel structures. For instance, the liquid-crystalline nanofilaments formed by Fmoc-Phe-Phe-Asp-OH in neutral pH could be employed as templates for the production of TiO_2_ nanofibers, which were reported to display better photocatalytic activity than TiO_2_ synthesized without template [143].

Cadmium sulfide (CdS) nanoparticles are noble metal-based materials that have been widely used in several technological applications owing to the suitable properties. For instance, the good resistance to corrosion, cost-effectiveness, low cytotoxicity, narrow band gap, and high photosensitivity, have propelled the use of these particles in areas ranging from biosensing and bioimaging to the development of photovoltaic cells and photocatalysis [144,145,146]. Moreover, the production of materials with well-defined arrays of nanoparticles has been of considerable interest to tune and improve the materials’ properties. In this sense, Banerjee et al. [147] developed peptide-based pH-responsive thermoreversible hydrogels with immobilized luminescent CdS nanoparticles. The authors demonstrated that the controlled deposition of the CdS particles on the gel fibres afforded definite green fluorescence arrays on the nanofibrillar structures, resembling small green threads. The immobilization was further accompanied by a blue shift of the emission spectra and photoluminescence band gap energy of the CdS nanoparticles, which opened the possibility of using peptide-based hydrogels as a suitable strategy to tune the materials’ optoelectronic properties without requiring changes of the particles size.

Although less reported in the literature, other materials can be incorporated to benefit peptide hydrogels in terms of structural and functional properties. Metals such as palladium (Pd) and platinum (Pt) are among them, with applications revolving around catalysis [148,149,150,151,152,153]. For example, Maity’s group developed a hydrogel from a tyrosine and tryptophan-based bolaamphiphile that was used as a template for the synthesis of Pd nanoparticles [148]. The nanofiber-stabilized particles were able to remove different amino acid N-protecting groups in the presence of NaBH_4_ in aqueous media [148]. On the other hand, Ghorbani-Choghamarani et al. used an arginine-based hydrogel, in which palladium particles coordinated with the amino acid residues, promoting aryl/alkyl halide cross-coupling reactions with 2-mercaptobenzothiazole or thiourea to obtain symmetric sulfides [149]. Regarding platinum, most studies also employ peptide bolaamphiphile-based hydrogels. These materials are used to modulate the in situ synthesis of ultrafine platinum nanoparticles capable of catalysing the hydrogen evolution reaction [151] and hydrogenations [152]. For instance, Kori et al. [151] reported the in situ synthesis of platinum nanoparticles in bolaamphiphile hydrogels that displayed excellent electrocatalytic activity in basic and neutral pH solution, as well as good stability for the electrochemical hydrogen evolution reaction, which has great potential for energy conversion applications. Featuring a markedly different hydrogelator both in size (20 amino acids) and structure, Wang’s group studied for the first time the assembly behaviour of a β-hairpin under the action of Pt (IV) ions, which were later reduced to nanorods. In this work, palladium was included for two purposes: to trigger peptide self-assembly and to confer catalytic activity to the platform. The electrocatalysis results of these nanostructures proved to be equivalent to that of commercial Pt/C [153]. Similarly, the same group demonstrated that the same peptide self-assembly into nanofibrils could be triggered in the presence of Cu^2+^ and be used as a template for the synthesis of CuS nanowires [154]. Moreover, the nanowires could produce a large temperature increase under NIR laser irradiation, endowing it as a potential nanomaterial for biomedical applications. Meanwhile, peptide hydrogel–copper sulfide (CuS) nanoparticle composites have been investigated in wound healing and optoelectronics [127,155]. Regarding the former, despite the skin having an impressive self-healing capability, it can be interfered by external factors, which, together with pathological factors, can lead to chronic wounds [156,157]. A bacterial infection is a common external factor that can delay the healing process, which can even lead to infection-related complications that include septicaemia or even death [158,159]. Further, the emergence of drug-resistant bacteria makes difficult the use of antibiotics that are commonly used to treat bacterial infections [160,161]. The ability of CuS to produce heat from light through the d-d energy transition and ROS is very promising in antimicrobial applications [162], especially when conjugated with antibacterial peptides. This is the premise of a recent work in which the well-known hydrogelator RADA16-I was combined with antibacterial peptides and CuS quantum dots [155]. This system has a contact-based antimicrobial matrix (hydrogel), hyperthermia agents (CuS quantum dots), and light-induced release of antimicrobial compounds, offering good perspectives in infection control and wound healing [155]. As previously mentioned, the incorporation of CuS nanoparticles into peptide hydrogels further enables the modulation of their optoelectronic properties. In this context, Palui et al. immobilized CuS NPs in a defined array in the nanofibrillar structure of hydrogels based on tripeptides with at least two phenylalanine residues [127]. The authors found a hypsochromic shift of the emission spectrum as well as a change in the band gap energy of the gel-embedded particles [127].

In fact, the control of supramolecular interactions is probably the most fascinating, but also the most challenging, aspect in the design of peptide-based composites. Its prediction would allow estimating the final properties and behaviour of these materials, making their practical application easier in several areas. Whether in the field of biomedicine, electronics, catalysis, or environmental technology, there is a noticeable lack of theoretical and computational studies that translate a more rational design and consider the components and their respective applications.

## 8. Conclusions

The addition of non-gelling components into supramolecular peptide hydrogels has undeniable potential in improving the materials—both structurally and functionally—for a variety of applications, ranging from optoelectronics to biomedicine. The characteristics and properties gained in the formulation of such composites result from the different networks formed by organic (peptide–lipid) and/or inorganic interactions (e.g., peptide–metal) and can be tuned according to composition and environment.

The collection of studies presented in this review makes it clear that there is still a long way to go in peptide-based composite hydrogels, highlighting the almost unexplored clay-containing materials. The first step is to further understand peptide self-assembly, non-classical phenomena (such as nanoparticle nucleation and growth), specific interactions, structure/properties relationship, and the mechanisms behind performance enhancement. This will provide the basis for filling the gap of knowledge regarding the medium- to long-term effects of these complex systems in several research areas.

Nevertheless, the attention of the scientific community is essentially focused on therapy and diagnostics, with controlled drug delivery in the spotlight. Most studies are still performed at a laboratory scale, so in vivo and long-term approaches are imperative to unravel the side effects and stability of the gels. Furthermore, to translate them into the clinics, synthesis protocols—for both LMWG and fillers—must be suitable for large-scale production.

Such challenges can only be surpassed by the synergistic work of several disciplines. Fortunately, more and more research groups are focusing their efforts on the field of peptide-based composites, which promise advances with great impact on society and the quality of human life.

## Figures and Tables

**Table 1:** Summary of the applications, advantages, and disadvantages associated with the peptide-based hydrogels loaded with the commonly used composites discussed in this review. The disadvantages can be transversal to other composites.

Composite	Applications	Advantages	Disadvantages
Liposomes/Niosomes	Drug/Gene delivery Cell culture	Delivery of unstable drugs Overcomes hydrogel and liposome limitations Improved therapeutic efficacy	Can be detrimental to gel’s mechanical properties
Magnetic nanoparticles	Drug delivery Cell culture Hyperthermia MRI	Synergy with magnetic hyperthermia Magnetoresponse MRI contrast	Requires screening functionalization to achieve co-assembly
Silica/Clay nanoparticles	Tissue engineering Wound healing	Improved biological and mechanical properties	Limited number of reported peptide-based gelators
Gold/Silver nanoparticles	Biosensing Drug delivery Catalysis	Low-cost sensors Synthesis in situ Facile synthesis and tunability Synergy with photothermia/photodynamic therapy	Heterogeneous dispersion Uncontrolled release Challenging reproducibility of in situ synthesis
Carbonaceous nanoparticles	Drug/Cell delivery Tissue engineering Catalysis Sensing	Improved network stability Reinforced mechanical properties Synergy with photothermia/photodynamic therapy Enhanced catalytic activity	Lacking studies of long-term cytotoxicity

**Table 2:** Summary of advancements with peptide LMWG-based magnetic hydrogels. 2-Naph: 2-(Naphthalen-6-yl) acetic acid; DA: Dopamine; Phe: Phenylalanine; PDA: polydopamine; PEG: poly (ethylene glycol); PAA: Poly (acrylic acid); Npx: Naproxen; Asp: Aspartate; Tyr: Tyrosine; ΔPhe: dehydrophenylalanine; met: Methionine; Cbz: Carboxybenzyl.

Nanoparticles	Hydrogel	Applications	Highlight	Reference
Fe_3_O_4_–DA-L-Phe-L-Phe-OH	2-Naph-L-Phe-L-Phe-OH	-	Magnetoresponse with small nanoparticle concentration	[77]
PDA–Fe_3_O_4_	H-Phe-L-Phe-OH	-	Co-assembly of different nanostructures into magnetic gels	[78]
Fe–PEG	Fmoc-L-Phe-L-Phe-OH	-	Supramolecular anisotropic magnetic gels	[73]
Fe_3_O_4_–PAA	Npx-L-Tyr-Z-ΔPhe-OH Npx-L-Asp-Z-ΔPhe-OH	MRI Magnetic hyperthermia	Dual T_1_/T_2_ MRI contrast	[79]
Fe_3_O_4_	2-Naph-L-Gly-L-Phe-L-Tyr-L-Asp-OH	Drug delivery	Magnetoresponse On-demand release	[80]
Fe–PEG	Fmoc-L-Phe-L-Phe-OH Fmoc-L-Arg-L-Gly-L-Asp-OH	3D scaffolds	Injectable Biocompatible Cell delivery	[81]
MnFe_2_O_4_–Au	Npx-L-Met-Z-ΔPhe-OH	Drug delivery	Photothermia-enhanced drug release	[82]
MnFe_2_O_4_–Citrate MnFe_2_O_4_–Lipid coating	Cbz-L-Met-Z-ΔPhe-OH	Drug delivery Hyperthermia	Low frequency AMF Enhanced drug release	[83]

**Table 3:** Summary of advancements with peptide-based metal nanoparticle–hydrogel composites. ADDA: 12-amino dodecanoic acid; Ar: Fmoc/Nap/Cbz; X^1^: phenylalanine/tyrosine/leucine/serine; Ac: acetyl; X^2^: different counterions.

Nanoparticles	Hydrogel	Applications	Reference
AuNPs AgNPs	H_2_N-ADDA-Phe-Phe-OH	Catalysis	[118]
AuNPs	Ar-Phe-X^1^	-	[107]
AuNPs	Fmoc-Phe-Glu-Lys-Phe-OH	Drug delivery Electrochemical sensing	[109]
AuNPs	Ac-Ile-Val-Phe-Lys-NH_2_	Catalysis	[110]
AuNPs AgNPs	Bile acid-dipeptide	-	[119]
AgNPs	Cl^− +^(H_3_C)_3_-Trp-CONH-C_16_H_33_ Cl^− +^(H_3_C)_3_-Tyr-CONH-C_16_H_33_ C_15_H_31_-CO-Val-Trp-COO^−^ Na^+^ C_15_H_31_-CO-Ile-Trp-COO^−^ Na^+^	Antibacterial activity	[108]
AuNPs AgNPs	Fmoc-Phe-Phe-OH	SERS sensing	[117]
AgNPs	X^2− +^(H_3_C)_3_-Trp-CONH-C_16_H_33_	Antibacterial activity	[112]
AgNPs	Nap-Phe-Phe-Cys-OH	Antibacterial activity	[113]
AgNPs	Ac-Lys-Ile-Val-Ala-Gly-Lys-NH_2_	Antibacterial activity	[114]
AgNPs	Nap-Lys (Nap)-ethyleneoxy-NH_2_	Antibacterial activity	[115]
AgNPs	Fmoc-Phe-Phe-Cys-Trp-Arg-OH	Catalysis Antibacterial activity	[116]

**Table 4:** Summary of advancements with peptide-based graphene–hydrogel composites. PDADMAC: poly diallyldimethylammonium chloride; PVP: polyvinylpyrrolidone; Amoc: *N*-anthracenemethyloxycarbonyl; Fc: ferrocene.

Carbon Material	Hydrogel	Applications	Reference
GO	Fmoc-Tyr-Asp-OH Fmoc-Phe-Asp-OH	-	[127]
GO	Fmoc-Phe-Phe-Phe-OH	-	[126]
GO Reduced GO GO/PDADMAC Reduced GO/PDADMAC Reduced GO/PVP	H_2_N-Val-Glu-Val-Lys-Val-Glu-Val-Lys-OH H_2_N-Phe-Glu-Phe-Lys-Phe-Glu-Phe-Lys-OH	-	[125]
Carbon nanotubes GO sheets Carbon nanohorns	H_2_N-Leu-Phe-Phe-OH	-	[138]
GO	Py-Gly-Ala-Gly-Ala-Gly-Tyr-OH	Drug delivery	[129]
Oxidized carbon nanotubes GO	Fmoc-Phe-OH/Fmoc-Tyr(Bzl)-OH Fmoc/Tyr-OH/Fmoc-Tyr(Bzl)-OH	Drug delivery	[131]
GO/TREN nanoparticles	Max8 peptide	Drug delivery	[130]
GO Reduced GO	Glutathione	Drug delivery	[128]
GO	H_2_N-Phe-Glu-Phe-Lys-Phe-Glu-Phe-Lys-OH	Cell delivery	[133]
Graphene quantum dots	Amoc-Phe-OH Amoc-Tyr-OH	Drug delivery Tissue engineering	[139]
GO	H_2_N-Phe-Glu-Phe-Lys-Phe-Glu-Phe-Lys-OH	Tissue engineering	[132,134]
GO	Fmoc-Ile-Glu-Ile-Glu-Ile-Glu-Ile-CONH_2_ Fmoc-Ile-Ile-Ile-Ile-Glu-Glu-Glu-CONH_2_ Fmoc-Ala-Glu-Ala-Glu-Ala-Glu-Ala-CONH_2_ Fmoc-Ala-Ala-Ala-Ala-Glu-Glu-Glu-CONH_2_	Catalysis	[136]
GO/Fc-Phe-OH	Fc-Phe-OH	Sensing	[135]

## Data Availability

Not applicable.
